# Protective treatments for copper alloy artworks: preliminary studies of sodium oxalate and limewater effectiveness against bronze disease

**DOI:** 10.1007/s11356-022-24107-0

**Published:** 2022-11-16

**Authors:** Giulia Monari, Monica Galeotti, Mauro Matteini, Barbara Salvadori, Roberto Stifanese, Pierluigi Traverso, Silvia Vettori, Paola Letardi

**Affiliations:** 1 CNR-IAS, Via De Marini 16, Genoa, Italy; 2 CNR-ISPC Via Madonna del Piano 10, Sesto Fiorentino, Italy; 3 OPD, Viale F. Strozzi 1, Firenze, Italy; 4 Florence, Italy

**Keywords:** Bronze disease, Nantokite, Conservation treatments, Oxalate, Experimental design, NdT techniques, Copper alloys, Heritage science

## Abstract

Nantokite (CuCl) locked inside subsurface micro-pits has been recognised as the driving force to the form of corrosion called bronze disease. The use of the traditional corrosion inhibitor benzotriazole is questioned because of toxicity. So there is a need for alternative conservation treatments. This work is focused on the experimental design to test the effectiveness of sodium oxalate followed by treatment with limewater to face bronze disease on outdoor bronzes. A number of foundry bronze coupons were exposed to weathering at Genoa Experimental Marine Station (GEMS) exposure site and sprayed twice a week with a 5% NaCl solution for the first 124 days. After 562 days of natural weathering, the patinas on coupons were characterised with non-destructive techniques (NDT) and the presence of nantokite was verified. We designed a workflow, as similar as possible to conservation treatments on real artworks, to test a 3% w/v sodium oxalate treatment with two different application times, with or without limewater, on the coupons. The effectiveness of the treatments was analysed by comparison of surface properties by several NDT measurements. A statistical approach and XRD measurements directly on the corroded bronze surfaces are suggested as an effective way to characterise and compare the overall behaviour of bronze disease treatments for conservation.

**Supplementary Information:**

The online version contains supplementary material available at 10.1007/s11356-022-24107-0.

## Introduction

Copper and copper alloys have been widely applied in a number of human activities from prehistory to nowadays: among the many different fields of application, cultural heritage objects deserve special attention. Many studies have addressed the complex mechanisms involved in the corrosion behaviour of archaeological bronzes (Robbiola et al. 1998). Atmospheric corrosion studies have a milestone in the special issue dedicated to copper patina formation (Graedel et al. 1987; Graedel 1987). The growing interest in outdoor bronze monuments allowed to recognise the more complex corrosion behaviour of copper alloys in atmospheric conditions than pure copper’s (Robbiola et al. 1993; Kong et al. 2018). Recent works focused on the role of zinc and tin compounds in the atmospheric corrosion of copper alloys (Chang et al. 2019, 2020). It is generally accepted that the growth of bronze patinas/corrosion layers in outdoor exposure (atmospheric corrosion) is mainly controlled by electrochemical reactions under thin electrolyte layers. Beyond cuprite—which is the main corrosion product—sulphates and chlorides are the most common compounds in the corrosion layer, with brochantite and antlerite in SO_2_-rich environments and copper trihydroxychlorides Cu_2_(OH)_3_Cl (such as atacamite) mainly in marine atmospheres (Di Carlo et al. 2017). Besides the role of alloying elements, the nature and properties of patinas are strongly influenced by pollutants and pH: this is the reason why the strong increase in rain acidity and pollution levels due to the Industrial Revolution strongly affected the surface layer of outdoor bronze monuments in the last century. Many studies have deepened the knowledge about the several different mechanisms giving rise to the complex structure of historical patinas (Robbiola et al. 1993; Krätschmer et al. 2002; Chiavari et al. 2007; Wu and Wang 2019; Chang et al. 2020); nonetheless, a unifying view is still lacking (Letardi 2021).

From a cultural heritage conservation point of view, one of the most harmful phenomena on copper alloys artworks is the so-called bronze disease (MacLeod 1981; Scott 1990; Bozzini et al. 2017). This form of localised corrosion is caused by the reaction of chloride ions with copper in the presence of oxygen and high humidity. Cuprous chloride CuCl (nantokite) is recognised as the key compound which can react with water and oxygen, giving rise to a cyclic self-sustaining reaction with production of hydrochloric acid (HCl), cuprite (Cu_2_O), and isomers of Cu_2_(OH)_3_Cl (atacamite and other polymorphs have been identified) (Di Carlo et al. 2017; Krivovichev et al. 2017). The result is the formation of pits with powdery green copper trihydroxychlorides, causing detrimental effects for the conservation of archaeological and outdoor copper alloy cultural heritage objects. The electrochemical driving forces of this active corrosion were highlighted (Bianchi and Longhi 1973; MacLeod 1981) and several papers addressed some peculiar aspects and suggested possible intermediate reactions (Bozzini et al. 2017). Nonetheless, the reaction path giving rise to this detrimental localised corrosion is not completely clear (Grayburn et al. 2015). Among the several factors highlighted, pH and ion concentrations play a key role on thermodynamic equilibria (Bianchi and Longhi 1973; Chase et al. 2007; Schindelholz et al. 2018); the kinetics of the pitting reaction must be carefully considered, too (MacLeod 1981; Schindelholz et al. 2018). Although nantokite is very slightly soluble, it hydrolyses in water to form cuprite (Cu_2_O) (MacLeod 1981; Grayburn et al. 2015):

$$2\mathrm{ CuCl}\hspace{0.17em}+\hspace{0.17em}{\mathrm{H}}_{2}\mathrm{O}\hspace{0.17em}\to \hspace{0.17em}{\mathrm{Cu}}_{2}\mathrm{O}\hspace{0.17em}+\hspace{0.17em}2\mathrm{ HCl}$$

and other copper compounds, with a reaction path strongly dependent on pH and on the activity of chloride ions (MacLeod 1981; He and Wang 2018). Acidic conditions have been shown to be the most detrimental in the context of bronze disease (Mazzeo et al. 2007; He and Wang 2018; Chang et al. 2020).

The complex surface layer growing on copper alloy heritage objects is the result of an evolving dynamic equilibrium between changing environments (because of excavation and post-excavation repository for archaeological objects or for the weather and climatic evolution in case of outdoor monuments) and the conservation treatments (cleaning and protection) (Letardi 2021). This makes it quite difficult to develop effective models. A deeper and deeper understanding of the several concomitant mechanisms (Kvashnina et al. 2007; Alfantazi et al. 2009; Schindelholz et al. 2018) has been growing, but a full comprehension is yet to be achieved.

From a phenomenological point of view, the key point is to keep heritage objects in a reasonably stable state, which can be obtained by conservation in a sufficient dry environment or by using inhibitors and coatings, to be renewed when necessary (Mazzeo et al. 2007; Cano and Lafuente 2013; Letardi 2021).

Among the corrosion inhibitors, benzotriazole (BTA) has been the most widely adopted for copper alloys by conservators, but despite the huge literature on this topic (Letardi 2021), a clear understanding of its efficacy for outdoor bronze monuments has not been reached. Its effectiveness seems to be affected by type and morphology of the corrosion patina and application method (Cano and Lafuente 2013). Important limiting factors, such as the tendency to change the colour of natural patinas, were pointed out: the more severe of them is its toxicity, which is the main reason for research on greener and healthier alternatives (Cano and Lafuente 2013; Letardi 2021).

Several challenges must be faced to screen a conservation treatment that adequately meets aesthetic, historical, and conservative issues (Matteini et al. 2016). During some conservation works carried out between 2006 and 2013, positive feedback was obtained, on the basis of some trials, with a treatment based on sodium oxalate solution followed by a limewater solution (Bellini et al. 2014; Matteini 2015; Matteini et al. 2016). The underlying idea is that sodium oxalate and atmospheric oxygen can react with the detrimental nantokite to form insoluble copper(II) oxalate CuC_2_O_4_ (moolooite) together with cupric oxide CuO (tenorite), as described assuming the following reaction:

$$4\mathrm{ CuCl}\hspace{0.17em}+\hspace{0.17em}2 {\mathrm{Na}}_{2}{\mathrm{C}}_{2}{\mathrm{O}}_{4}\hspace{0.17em}+\hspace{0.17em}{\mathrm{O}}_{2}\hspace{0.17em}\to \hspace{0.17em}2\mathrm{ Cu}{\mathrm{C}}_{2}{\mathrm{O}}_{4}\hspace{0.17em}+\hspace{0.17em}2\mathrm{ CuO}\hspace{0.17em}+\hspace{0.17em}4\mathrm{ NaCl}$$

The resulting sodium chloride (NaCl) is highly soluble and can be easily removed by rinsing, while moolooite and tenorite are insoluble stable products commonly observed on bronze patinas (Dolcini 1988; Matteini et al. 1992; Marabelli and Mazzeo 1993; Letardi et al. 2004; Mazzeo 2005) characterised by chromatic compatibility with the usual—pale greenish and dark brownish—natural patina colours (Matteini et al. 2016). Moolooite is often detected on copper alloy artworks’ patinas exposed in outdoor conditions, as a result of interactions between the alloy and organic materials (Dolcini 1988; Matteini et al. 1992; Marabelli and Mazzeo 1993; Letardi et al. 2004; Mazzeo 2005). Furthermore, bio-based treatments have been tested to passivate metal artworks producing copper oxalate (Mazzeo et al. 2007; Joseph et al. 2011, 2012; Albini et al. 2018). Therefore, the treatment is supposed to inhibit bronze disease triggered by nantokite.

On the other hand, the limewater solution involves a pH change on the bronze surface, from acidic (condition favourable to pitting corrosion) to basic (unfavourable), initially due to calcium hydroxide and afterward to insoluble calcium carbonate (Mazzeo et al. 2007). The use of limewater to adjust pH and alkalinity of the corroded substrate so that further corrosion can be inhibited was considered in previous works (Mazzeo et al. 2007; Bruni 2017). A 5% concentration applied on copper coupons with 80-year-old natural brochantite patina was found ineffective with respect to the traditionally used benzotriazole, while the results on bronze coupons with 1-year natural patina in a marine environment were the opposite (Mazzeo et al. 2007).

In the last few decades, several laboratory projects addressed the issue of more effective treatments for the conservation of outdoor bronze artworks, with a wide range of more or less simplified choices for the tested coupons (Salvadori et al. 2018). None of these treatments has yet been largely recognised as a better solution in conservation works compared to the widely adopted paraloid/incralac and/or waxes (with or without the unhealthy BTA). Nonetheless, they allowed a growing awareness of the mutual influence of the many parameters that may affect in-service behaviour of treatments on outdoor bronzes, such as patina composition and morphology, and the detailed application protocols. The lack of well-assessed and standard methods to test protective treatment effectiveness for surfaces with complex patinas in different environments is one of the main shortcomings towards innovative methods in outdoor bronzes’ restoration and maintenance (Letardi 2021).

**Fig. 1:**
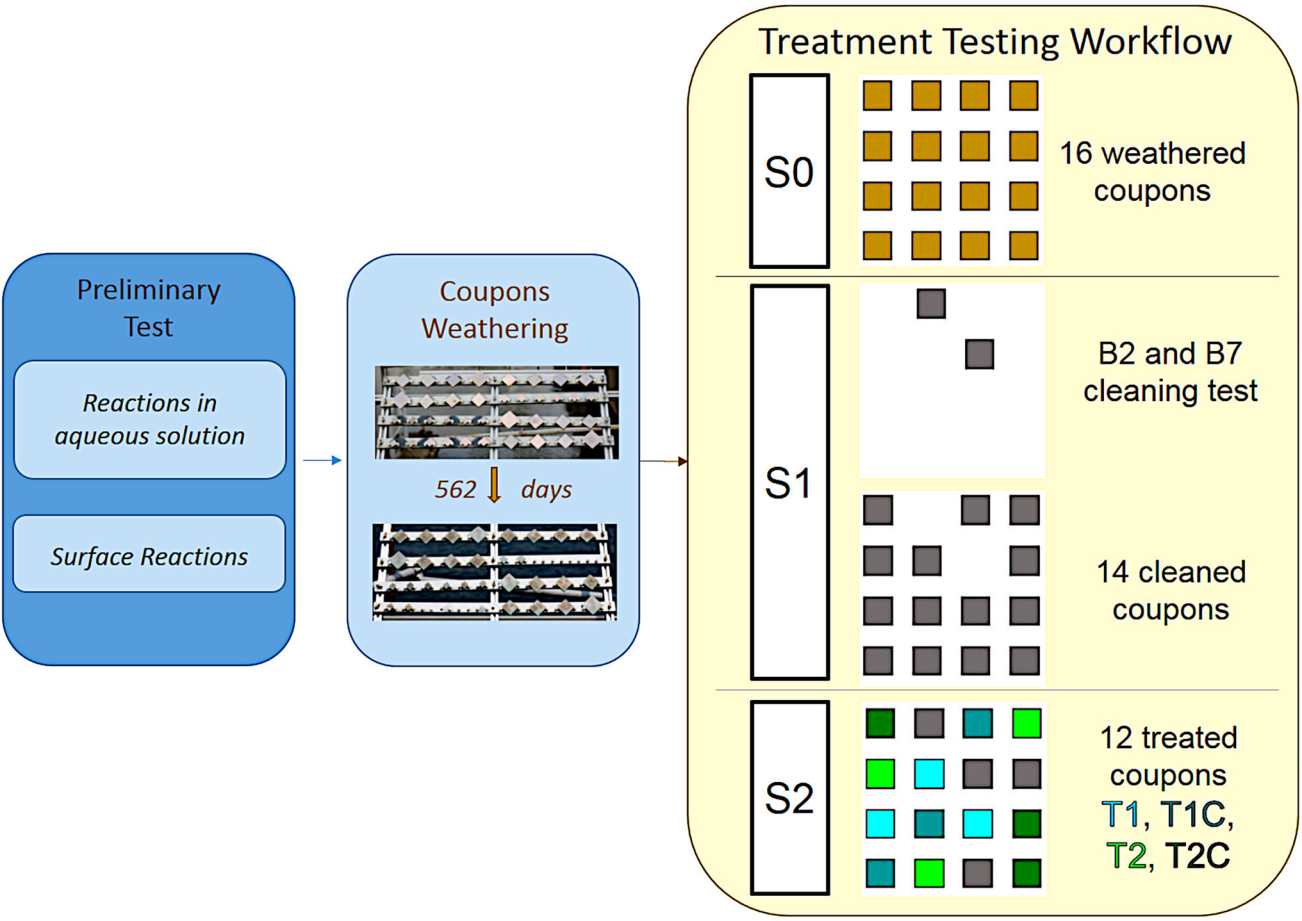
Synoptic diagram of the experimental work described. After some draft tests to check reaction of nantokite with sodium oxalate (preliminary test), sixteen bronze coupons were weathered at Genoa Experimental Marine Station (GEMS) about eighteen months (coupon weathering). We then adopted the following treatment testing workflow on the weathered coupons: (S0) NDT measurements on the 562-day weathered coupons; (S1) cleaning test on two of them, followed by cleaning of the remaining fourteen coupons with the selected methodology and their NDT characterisation; (S2) application of the sodium oxalate treatment with different times (T1, T1) with possible addition of limewater (T1C, T2C) and their NDT characterisation

To add a piece of information towards more successful conservation treatments of outdoor bronze monuments, we made some preliminary experiments to better characterise the behaviour of sodium oxalate and limewater treatments (NOX-LW) to tackle bronze disease. A detailed analysis of the manifold equilibrium path involved in the several chemical-physical parameters affecting the treatment and its application method on heritage surfaces was beyond the scope of this research. So we first made some qualitative tests on reaction equilibria; then we focused our attention on the design and testing of an experimental method suitable to check treatment results on surfaces as similar as possible to outdoor bronzes’ ones (Monari 2019). This paper reports a novel experimental workflow to test conservation treatments tailored to better focus on the complex outdoor bronze surfaces (Fig. 1); marine-weathered coupons were prepared; their surface properties were characterised by means of several non-destructive techniques before and after cleaning/treatments, and compared with the properties measured on artworks. The unevenness of surface properties is discussed along with the analytical methodology adopted to properly deal with the large variability of the measurements. Moreover, a first discussion of NOX-LW treatment performance at different concentrations and application times is presented. Further studies are suggested to deepen treatments’ application and effectiveness after weathering.

## Materials and methods

### Preliminary tests on possible reactions involved

Available reagent grade chemicals have been used and checked by XRD. Copper oxalate (moolooite, CuC_2_O_4_·0.4H_2_O) was synthesised in the laboratory (D'Antonio et al. 2007) to acquire an XRD reference diffractogram.

Powder XRD data acquired to check the available reagent-grade nantokite highlighted the low stability of this compound at ambient conditions, with easy evolution to eriocalcite (CuCl_2_∙2H_2_O). Thus, nantokite was synthesised in the lab as follows: CuCl_2_ (4.87 g, Carlo Erba, Italy) was added to 500 ml boiling deionized water in continuous stirring. Cu powder (< 200 mesh, Sigma Aldrich, Italy) was then slowly added (10.3 g) to the CuCl_2_ solution until an insoluble material appeared. The stirring was then stopped and the Cu powder quickly precipitated at the bottom of the flask. The supernatant was separated from the Cu powder precipitate by a vacuum pump, transferred in a new flask, and left to sediment by gravity at room temperature. After 24 h, the supernatant was clear and was removed by a vacuum pump and discarded, whereas the precipitate was left in the flask. After 24 h at room temperature, the precipitate, completely dried, was analysed by powder XRD and identified as nantokite (Fig. SI-1).

To roughly check the main reaction products between nantokite and sodium oxalate ((COONa)_2_), the following tests were performed in deionised water: a) addition of sodium oxalate to a nantokite-saturated solution; b) addition of nantokite to a sodium oxalate–saturated solution. The precipitated products obtained were collected and analysed by powder XRD (Fig. SI-2).

Furthermore, to test the reaction on surfaces, nantokite was also synthesised over copper plates: 3 × 3 cm coupons were polished with grit 800 SiC paper, then immersed for 5 h in 50 ml deaerated 37% HCl at 50 °C; HCl excess was then removed by absorption on paper, and complete drying was obtained under N2 continuous insufflation. The formation of nantokite was verified by XRD measurements on the coupons surface (Fig. SI-3).

A sodium oxalate poultice was applied as a thick layer (supersaturated solution) over one of the prepared coupons and kept there for 5 days at room temperature and then removed (a). Another coupon was immersed for 17 h in a 5% w/v sodium oxalate solution (b). A third coupon was immersed for 17 h in deionised water (c). The results were checked by repeating XRD measurements on coupon’s surface (Fig. SI-3).

### Weathered coupons

Sixteen 5 × 5 cm quaternary bronze (Cu 85%, Sn 5%, Pb 5%, Zn 5%) coupons, casted by Fonderia Ciglia e Carrai S.r.l. (Florence, Italy), were polished with sandpaper up to 1200 grit size and exposed in *Genoa Experimental Marine Station* (GEMS) (Stifanese et al. 2018) following ISO 8565 standard for 562 days (a bit more than 18 months). In order to enhance corrosion by chlorides, the coupons were sprayed twice a week with a 5% w/v NaCl solution for the first 124 exposure days (Letardi et al. 2017; Letardi 2021).

### Treatment testing workflow

**Fig. 2:**
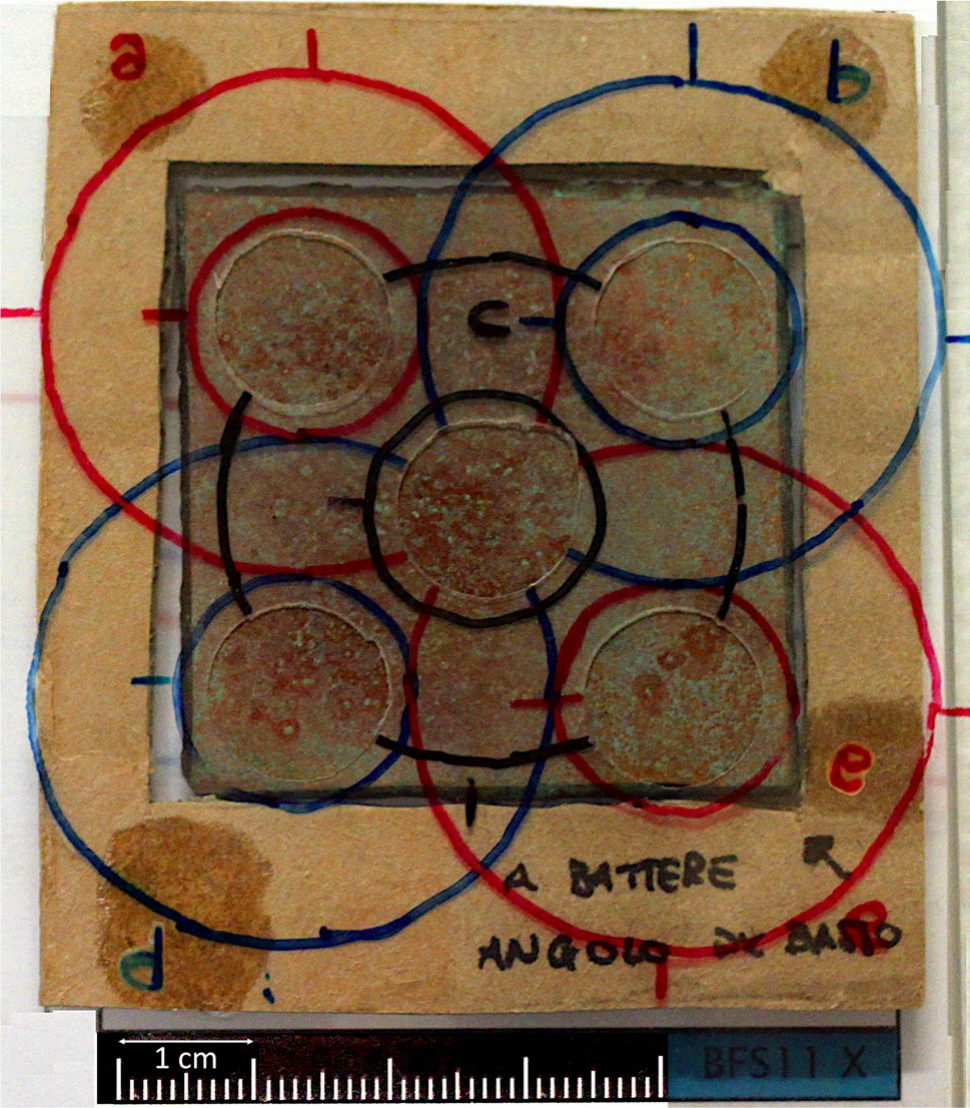
Measurement frame with the five areas *a*, *b*, *c*, *d*, and *e* positioned over coupon 11. The black arrow on the bottom right points to the number engraved on the backside of the coupon

The test method to be carried out on coupons was designed to follow the workflow in usual conservation treatment as much as possible: first of all, the conservator has to define a proper cleaning method in order to remove detrimental, unstable, or disfiguring surface products from the corroded bronze surfaces and then applies a protective treatment. To this end, we examined the sixteen coupons weathered at GEMS to characterise their surface properties through three experimental phases (Fig. 1):

- S0: initial state of coupons after the 18-month weathering in marine atmosphere
- S1: cleaned coupons
- S2: treated coupons
  All the sixteen coupons were preliminary marked on the lower right back side angle with a unique identification number 1 to 16. Five 8-mm diameter monitoring areas for each coupon were defined and labelled *a*, *b*, *c*, *d*, and *e*. A frame perfectly fitting to the shape of the coupons and supporting a PVC transparent sheet was created with holes corresponding to the five selected areas to repeat measurements on the same spots throughout all the experimental phases (Fig. 2). The frame was positioned over the coupons in a fixed orientation with respect to the marked identification number; this made it possible to uniquely identify the measuring areas on each coupon.
  The evolution of chemical and physical surface properties was analysed with a number of non-destructive analytical techniques through the three experimental phases (Table 1). The analytical techniques were chosen to allow a more straightforward comparison with measurements on real objects. They provide a visual-aesthetic evaluation and a chemical, morphological and protective characterisation of surfaces. Measurements with all the analytical techniques adopted on all five areas of each sample were infeasible due to measurement acquisition and analysis time required. The sampling scheme adopted is reported in Table 1. A special attention was paid to obtain statistically significant datasets.
  Characterisation at the S0 phase (coupons after marine weathering) was aimed at checking patina composition, morphology, and unevenness, with a special focus on the presence of nantokite. Two samples were then selected for cleaning tests to assess the more appropriate cleaning methodology. The selected cleaning method was then applied on the remaining 14 samples. Characterisation at the S1 phase (cleaned coupons) aimed at comparing patina composition, morphology, and unevenness with the S0 phase, with a special focus on the presence of nantokite. Afterwards, available samples were divided into five statistically equivalent groups for the NOX/LW treatment test: two coupons were left untreated (T0) and the remaining 12 coupons were used to test:
- the sodium oxalate treatment only, with two different application times (T1, T2) (3 + 3 coupons);
- the sodium oxalate treatment followed by the limewater treatment at the same two application times (T1C, T2C) (3 + 3 coupons).

**Table 1:** Overview of non-destructive techniques (NDT) measurements on the available coupons (S0, after 18 months weathering; S1, after cleaning; S2, after treatment)

NDT	S0	S1	S2
Microscopy	✔	✔	✔
Colour	✔	✔	✔
Thickness		✔	✔
Roughness		✔	✔
FTIR	2 ×	2 ×	2 ×
XRD	B2 (a, b, d, e) B7 (a, b, d, e)	B2 (a, b, d, e) B7 (a, b, d, e) B1 a B3 c B4 b B9 b B15 c	B1 a B3 c B4 b B9 b B15 c

✔ measurements on the five areas a, b, c, d, e on all the coupons

2 × measurements on 2 selected areas on all the coupons

Characterisation at the S2 phase (treated coupons) was aimed at comparing patina composition, morphology, and unevenness with the S1 phase, with a special focus on nantokite oxidation/conversion.

**Fig. 3:**
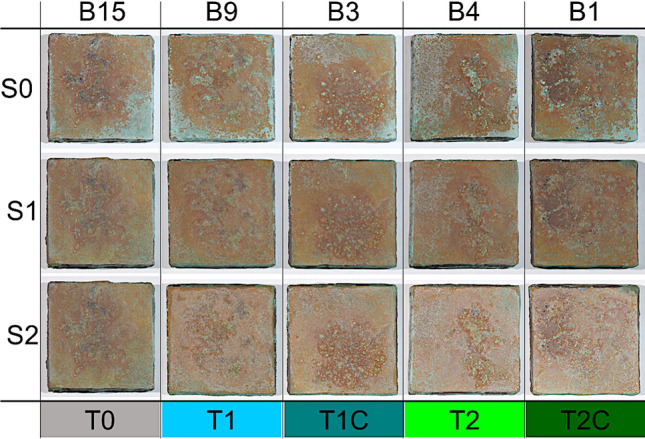
Comparison of the photographic documentation through the three experimental phases (S0, weathered coupons; S1, cleaned coupons; S2, treated coupons) of one coupon for each group (see Table 2 for treatment labels)

Figure 3 shows one coupon for each group through the three experimental steps considered.

### Analytical techniques

#### Microscopy

A morphological study of coupons with low magnification was performed by a Stemi2000 Zeiss Stereo Microscope equipped with a camera. The images acquired were then organised using ACT 1 Software. The superficial characteristics were also documented using a Dino-Lite Microscope Scalar DG-2A with × 25–200 magnification.

#### Colour

Standard colour measurements, according to the CIEL*a*b* colour reference space, were acquired with a portable spectrophotometer Minolta CM-700d [8 mm diameter measurement area, SCI/SCE, 360–700 nm, illuminant D65, 10° observer]. An average of three measurements was made for each point. The colour difference Δ*E* of each group before and after the treatments was calculated. The change in a* colour coordinate was also used as a rapid and efficient marker to characterise the evolution of the aesthetic conditions of patinas.

#### TR-FTIR

FTIR spectra were acquired with a portable Alpha Bruker spectrometer equipped with a camera and operating in total reflection mode (TR-FTIR), range 7000–375 cm^−1^, resolution of 4 cm^−1^, 128 scans, 6-mm measuring spot. The IR spectra were processed using OPUS software. The spectra were acquired on two areas for each coupon selected according to colour value measured at the S0 phase: on each coupon, the two areas with the lowest and the highest values of the a* colour coordinate were chosen to be monitored through the experimental steps S0 to S2.

#### XRD

X-ray diffraction was performed using a X’Pert PRO PANalytical equipment with radiation Cu Kα1 (*λ* = 1545 Å), operating at 40 kV, 30 mA, investigated range 3° < 2*θ* < 70°, time per step 60 s, increment 0.03°. XRD diffractograms were collected directly on coupons (in situ analysis) with a measurement area around 1.5 cm^2^. Samples B2 and B7 were characterised at the S0 and S1 phases to check the cleaning procedures (Table 1). One monitoring area was selected on B1, B3, B4, B9, and B15 to compare the surfaces before and after the treatments (Table 1). Coupons B3 and B9 were also analysed with a diffractometer Bruker New D8 “Da Vinci”, radiation Cu Kα, operating at 40 kV, 40 mA, to deepen the analysis at the S2 phase for the identification of moolooite by investigating the range 10° < 2*θ* < 60°, with time per step 768 s, increment 0.02°.

To monitor the variation of the identified mineralogical phases through the three experimental steps (Table 1), the counts over background for a selected XRD-peak max were used as a qualitative marker of the amount of the corresponding mineralogical phase in the patina layer. Namely, the peak at 36.4° was used for cuprite, the one at 28.5° for nantokite, while copper hydroxychlorides were monitored with the peak at 17.6° (atacamite) and 16.2° (atacamite + clinoatacamite). The peak at 22.9° was used to monitor moolooite and the one at 13.6° for the wheatleyite (Na_2_Cu(C_2_O_4_)_2_ ∙2H_2_O).

#### Thickness

Patina thickness on the coupons was measured by a LEPTOSKOP 2042 thickness gauge with ∅ = 3 mm measurement area.

#### Roughness

Surface roughness of coupons was measured by a SURFTEST SJ-210 (*R*_a_ =  − 200 µm to + 160 µm).

### Cleaning method

The cleaning procedure for the outdoor marine-weathered coupons was defined in a way as close as possible to the methods usually adopted for real artwork conservation: this is considered a paramount step in order to mimic the surface layer to be treated in conservation practice. Namely, cleaning tests were performed by a conservator on two coupons (B2, B7) selected as representative of the variability of the alteration patinas formed on the different specimens. Indeed, at the end of the natural weathering (step S0), a greenish thin homogeneous patina covered sample B2, while sample B7 was characterised by a considerable amount of greenish corrosion products unevenly distributed. Cleaning outcome was judged by the conservator and checked by observation under optical microscope as the minimum intervention to remove powdery and disfiguring corrosion products, with the same criteria adopted on artworks. The conservator then cleaned the remaining fourteen coupons with the selected procedure, which consisted in the following:

- cleaning with organic solvent (acetone and alcohol 1:1);
- soft mechanical cleaning with a cut bristle brush;
- local action of scalpel where corrosion products were more difficult to remove.

### Treatments

The sodium oxalate treatment (NOX) was applied following the previous experience on outdoor bronze monuments (Bellini et al. 2014; Matteini 2015; Matteini et al. 2016). A flat bristle brush soaked with a sodium oxalate solution was used to fix Japanese paper on the coupons’ surface; then a cellulose poultice compress soaked in the same sodium oxalate solution was applied over the Japanese paper and wrapped with a plastic foil to avoid drying out. The whole compress was removed after a defined amount of time and the surface was left to dry. The following day, the surface was rinsed with deionised water to remove soluble compounds and left to dry again. The limewater (LW) treatment was applied the day after rinsing in order to allow a complete drying: Japanese paper was laid over the surface and a cellulose poultice compress soaked with limewater (pH = 10) was applied for 1 h and then removed. To test the sodium oxalate and limewater treatments on the fourteen available coupons, we focused our attention on the application time of 3% w/v (COONa)_2_ (pH = 7) sodium oxalate (8 h for treatment T1 and 16 h for T2 one) and comparison of results with (T1C, T2C) or without (T1, T2) limewater. In the end, three coupons were available for each of the four treatments, as summarised in Table 2. Two coupons were left untreated (T0) as reference.

**Table 2:** Combination of sodium oxalate 3% w/v (NOX) and limewater (LW) treatments under investigation (see text for treatment application details) with corresponding labels. Reference untreated coupons are labelled T0

Treatment label	NOX application time	LW application time
T0	-	-
T1	8 h	-
T1C	8 h	1 h
T2	16 h	-
T2C	16 h	1 h

## Results

### Weathered coupons—S0

The sixteen coupons weathered at GEMS (step S0) were characterised to evaluate the aesthetic and chemical properties of patinas, the variability between coupons, and the formation of copper chloride within the surfaces.

**Fig. 4:**
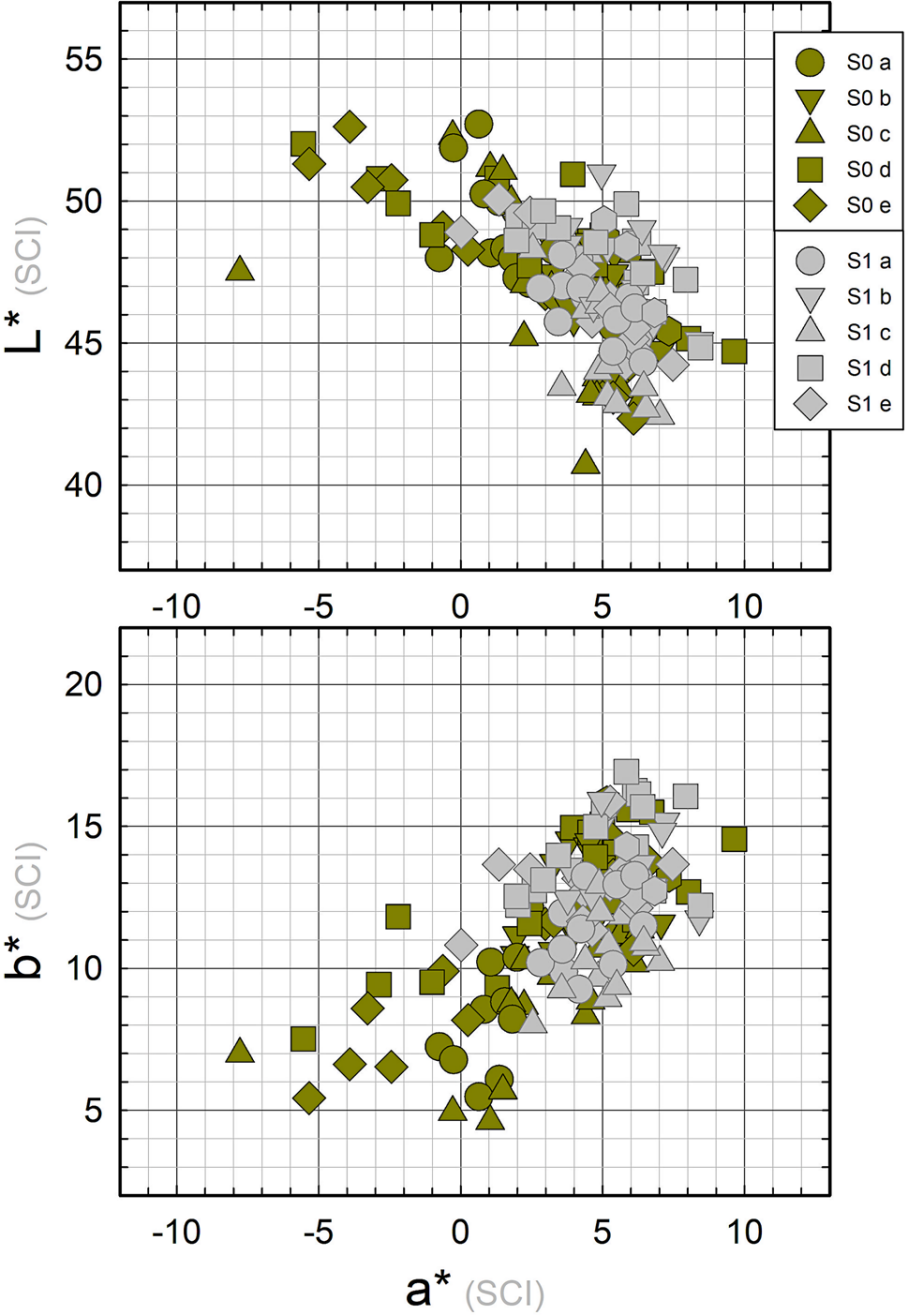
Colour data measured on the five monitoring points (see Fig. 2 for point labelling) for all the 16 coupons after weathering (S0) and for the 14 cleaned coupons (S1)

Results of colour measurements are reported in Fig. 4. The strong unevenness of coupons’ visual appearance is well described by the L*a*b* large value distribution, which is commonly observed on outdoor monuments, too. The average value of the 80 colour measurements was [L* = 47 ± 3; a* = 3 ± 3; b* = 11 ± 3].

**Fig. 5:**
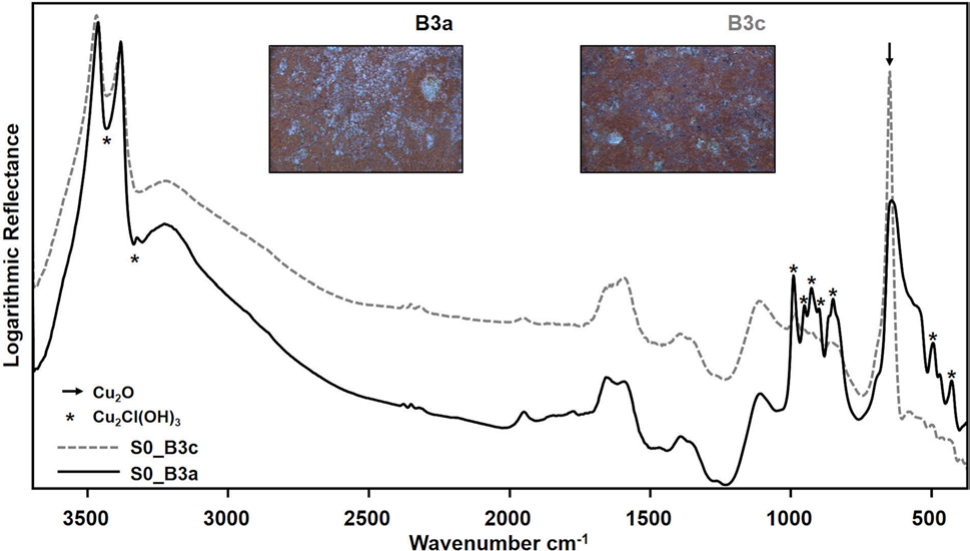
IR spectra at S0 measured on coupon B3 on the two areas with lower (B3a) and higher (B3c) a* colour coordinate. Stereomicroscope images of the same two areas are also reported. Asterisks mark the typical vibrations of copper hydroxychloride, while the arrow indicates the *ν*_s_(Cu–O) vibration of cuprite

On all the coupons at the S0 phase, TR-FTIR spectra showed corrosion products typically found on copper alloys after weathering in the marine atmosphere (Di Carlo et al. 2017), namely cuprite and copper hydroxychlorides (Fig. 5). Indeed, FTIR spectra of the green areas are dominated by the *ν*(OH) and *δ*(Cu–O–H) vibrations of copper hydroxychlorides, which fall in the regions 3600–3200 cm^−1^ and 1000–700 cm^−1^, respectively (Martens et al. 2003; Mendoza et al. 2004; Núñez et al. 2005; Šatović et al. 2010; Di Carlo et al. 2017; Buse et al. 2019; Ingo et al. 2019). The strong peak of *ν*_s_(Cu–O) at 648 cm^−1^ and the shoulder at 695 cm^−1^ on the red areas are attributed to copper(I)oxide, cuprite (Martens et al. 2003; Mendoza et al. 2004; Šatović et al. 2010).

**Fig. 6:**
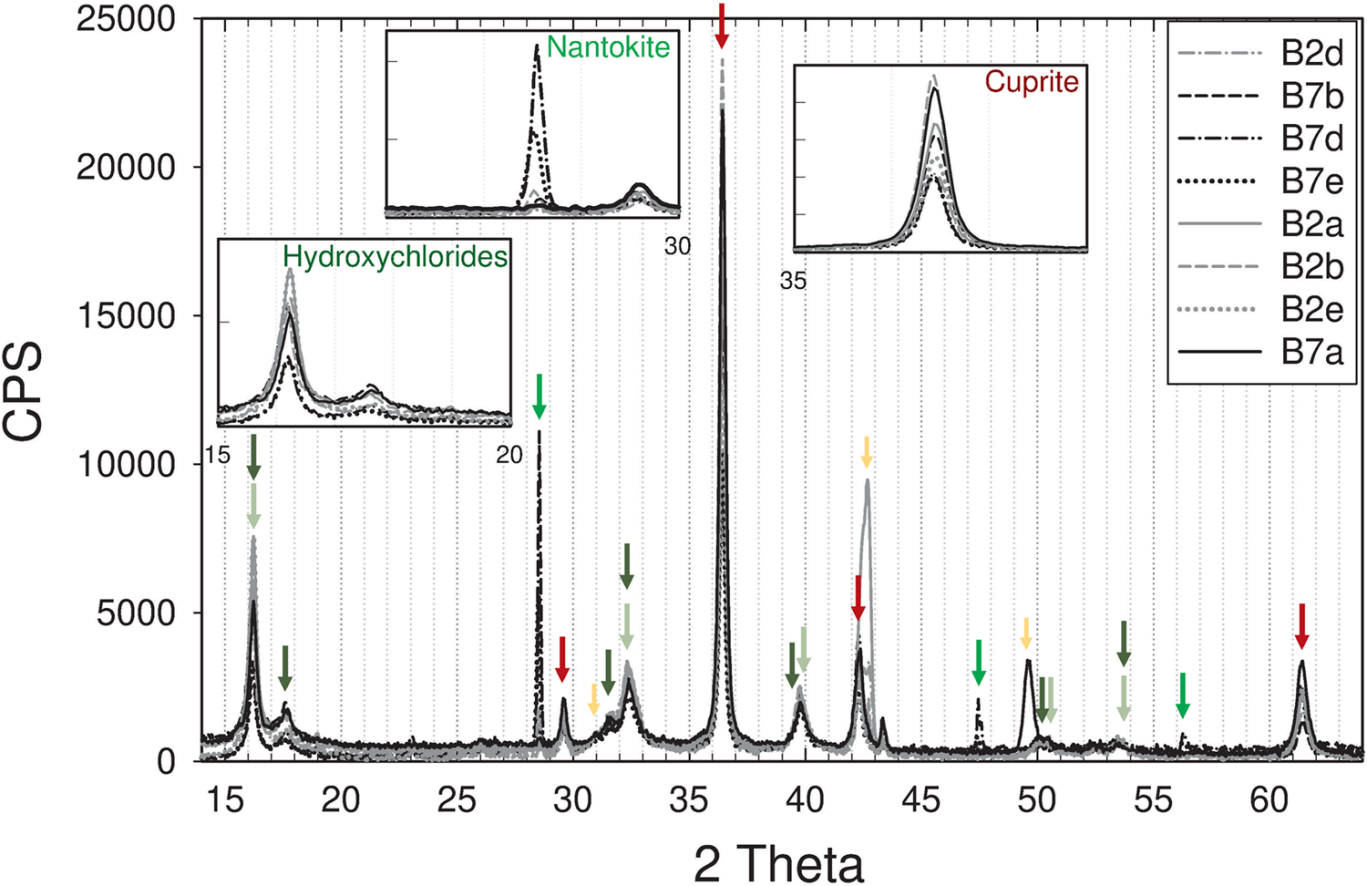
XRD spectra acquired on the eight monitoring areas analysed at S0 (see Table 1). Arrows point to the peaks used to identify the different phases: cuprite (red); nantokite (bright green); hydroxychlorides (atacamite in dark green; clinoatacamite light green). The yellow arrows correspond to peaks from the bronze alloy

XRD patterns on coupons B2 and B7 (Fig. 6) confirmed cuprite and copper hydroxychlorides as main patina components (Chmielová et al. 2003; Núñez et al. 2005; Di Carlo et al. 2017; Masi et al. 2017; Ingo et al. 2019), while nantokite was clearly identified on four of the measured points (Table 1). The large variation of nantokite’s counts—on the eight monitoring areas considered—points out to its localised formation and uneven distribution within the patina.

### Cleaned coupons—S1

At the S1 phase, the main goals of the measurements were to compare coupons’ surface properties with the ones at the S0 phase and to characterise the statistical distribution of measured values.

Colour data on the fourteen cleaned coupons are shown in Fig. 4. A more homogeneous distribution of chromatic coordinates than the one obtained at phase S0 is clearly visible, along with a reduction of the green component: the overall average colour value is [L* = 47 ± 2; a* = 5 ± 2; b* = 13 ± 2].

**Fig. 7:**
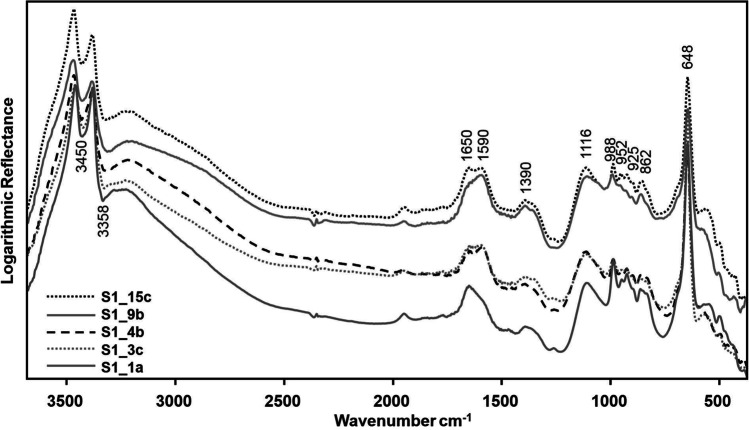
IR spectra measured on the five representative coupons B1, B3, B4, B9, and B15 on areas *a*, *c*, *b*, *b*, and *c*, respectively, collected before the chemical treatments

The partial removal of the green corrosion products caused the exposure of the underlying cuprite patina, as highlighted by TR-FTIR spectra (Fig. 7), which show the decrease of *ν*(OH) and *δ*(Cu–O–H) vibrations of copper hydroxychlorides within the 3500–3200 cm^−1^ and 950–800 cm^−1^ range, respectively (Martens et al. 2003; Mendoza et al. 2004; Núñez et al. 2005; Šatović et al. 2010; Di Carlo et al. 2017; Buse et al. 2019; Ingo et al. 2019) and the increase of *ν*(Cu–O) vibration around 650 cm^−1^ typical of cuprite (Martens et al. 2003; Mendoza et al. 2004; Šatović et al. 2010).

**Fig. 8:**
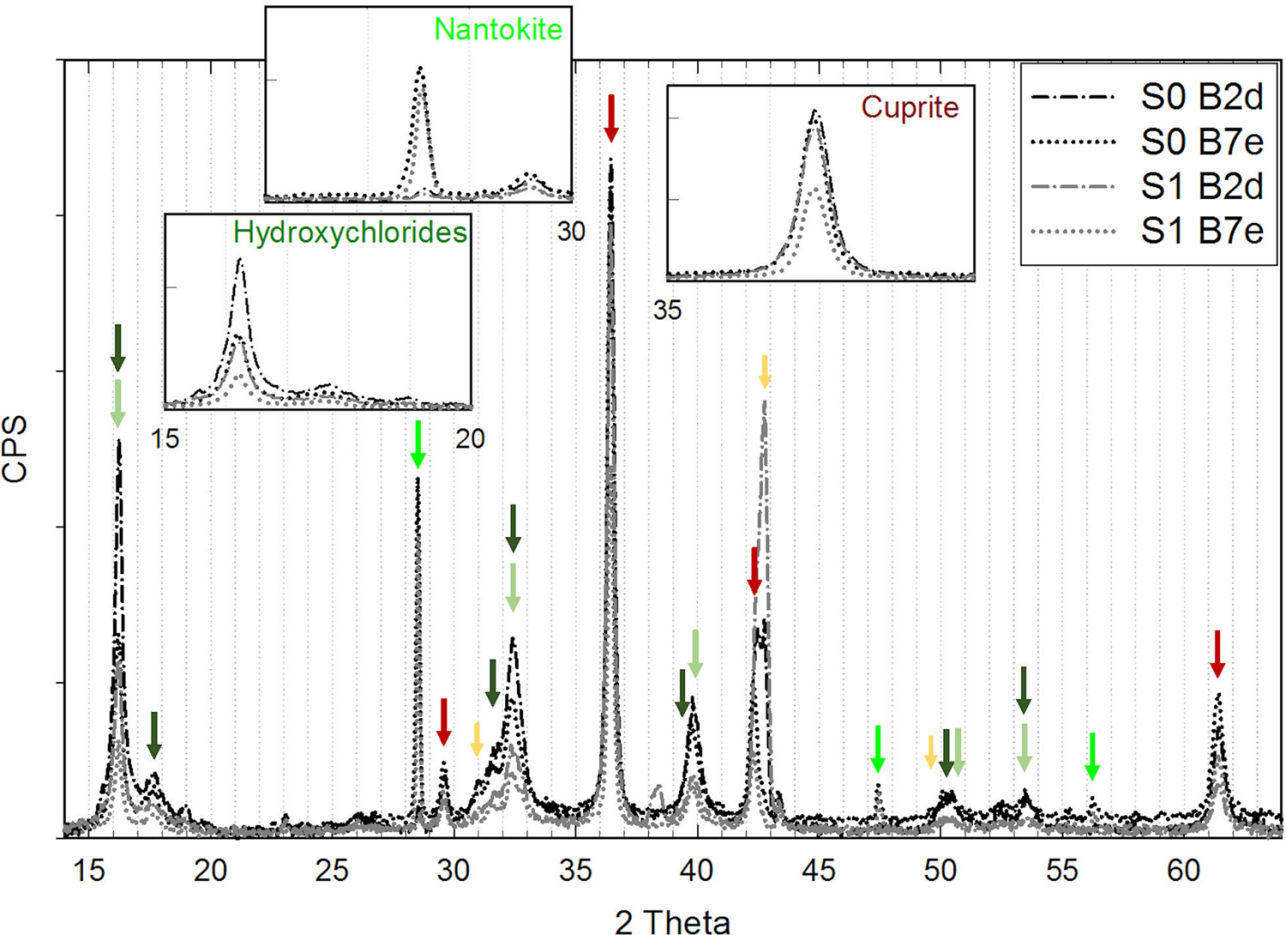
XRD pattern obtained after selected cleaning on coupons B2 and B7 (arrows as in Fig. 6)

The comparison of XRD data before and after the selected cleaning methodology on coupons B2 and B7 (Fig. 8) highlighted a strong reduction of copper hydroxychloride reference peak intensity on both coupons. On B2d a very slight reduction of cuprite is obtained while nantokite is characterised by a reduction of the same order of hydroxychlorides. On B7e, a greater reduction of the cuprite monitoring peak and a lower one for the nantokite monitoring peak is obtained. Monitoring peak counts on cleaned areas of B1, B3, B4, B9, and B15 show similar variability for cuprite and copper hydroxychlorides, while it is almost double for nantokite: this result again attests the more localised formation and uneven distribution of CuCl within the patina.

Roughness measurements provide an overall average value of Ra = 2.1 ± 0.6 µm, even though a bimodal distribution can be identified by a careful analysis: 65/70 cleaned areas have an average value of Ra = 1.9 ± 0.4 µm, while the other 5/70 are characterised by an average Ra = 3.5 ± 0.5 µm.

The overall average value of thickness data is 10 ± 4 µm. A more detailed analysis shows a multimodal distribution: 47/70 areas are characterised by an average thickness of < *s* >  = 11 ± 2 µm, with two smaller groups with < *s* >  = 18 ± 2 µm and < *s* >  = 5.9 ± 0.4 µm.

The large variability of measured surface properties calls for a careful grouping of the available coupons in order to avoid improper bias for the different treatment testing. To this end, the five XRD-monitored coupons were at first distributed into the five groups T0-T1-T1C-T2-T2C. The other available coupons were then distributed among the groups and the average value of the colour-roughness-thickness for each group at S1 phase computed: the distribution was modified until those average values could be considered the same within the experimental uncertainties for all the five groups.

### Treated coupons—S2

After the treatments, a significant amount of wheatleyite—a water soluble rare sodium cupric hydrated oxalate (Rouse et al. 1986)—was clearly identifiable on coupons. It was then decided to make a further rinsing with deionised water on coupons belonging to T1 and T2 groups.

At the S2 phase the main goals of measurements were to compare coupon surface properties with the ones at S1 and to examine the effects of the different NOX-LW application methods.

**Fig. 9:**
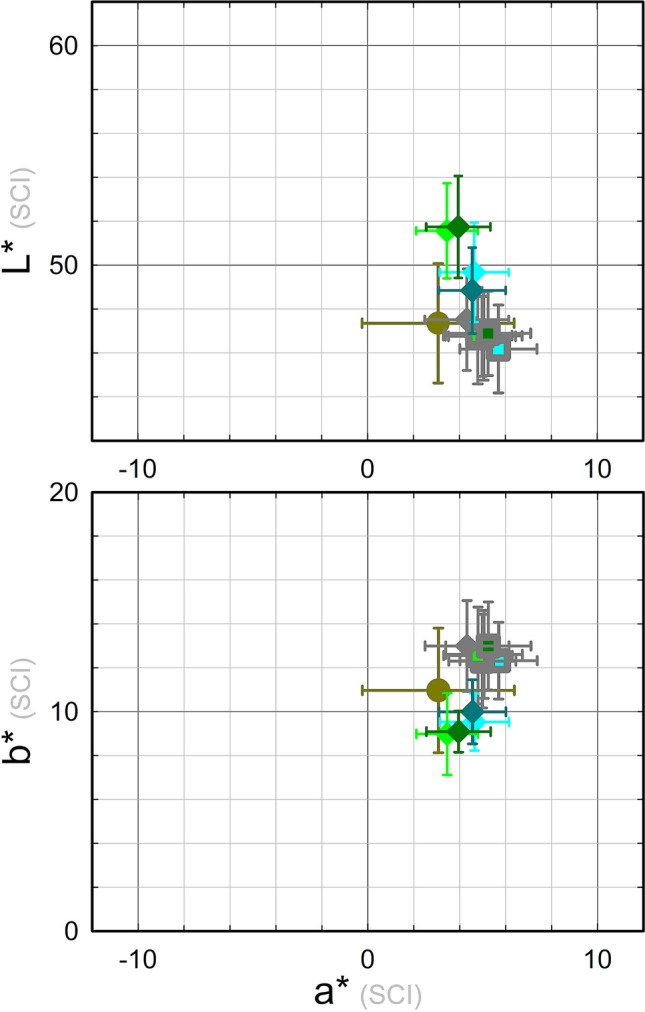
Average colour values obtained at the different experimental phases considered for the treatments under investigation: S0 (dark yellow); S1 (grey error bars); S2 (error bar colour according to treatment, as listed in Table 2)

L*a*b* average values are reported in Fig. 9 and colour differences Δ*E* with respect to S1 are reported in Table 3. The Δ*E* values overcome the threshold of eye sensibility (Δ*E* ~ 3) for all except T1C treatment. The colour trend is the same for all the application methods considered: an increase of the L* (bleaching) and a decrease of chromatic parameters more pronounced for b*.

**Table 3:** Average colour differences before (S1) and after (S2) treatment. For each treatment, 15 measurements were averaged

Treatment	ΔL*	Δa*	Δb*	Δ*E* S2-S1
T1	3.5 ± 0.7	− 1.1 ± 0.5	− 2.8 ± 0.8	5 ± 1
T1C	2.1 ± 0.8	− 0.4 ± 0.4	− 2 ± 1	3 ± 1
T2	5 ± 1	− 1.3 ± 0.6	− 4 ± 1	6 ± 2
T2C	4.8 ± 0.9	− 1.3 ± 0.7	4 ± 1	6 ± 1

The bleaching is greater after 16 h of sodium oxalate application (T2, T2C) than after the shorter application (T1, T1C) with barely larger ΔL* without LW. A general tendency towards a bluer hue can be observed for all treatments, to a greater extent for the longest sodium oxalate application, while a slight tendency towards greener hue is observed for all but T1C, which is characterised by a negligible modification of a*.

**Fig. 10:**
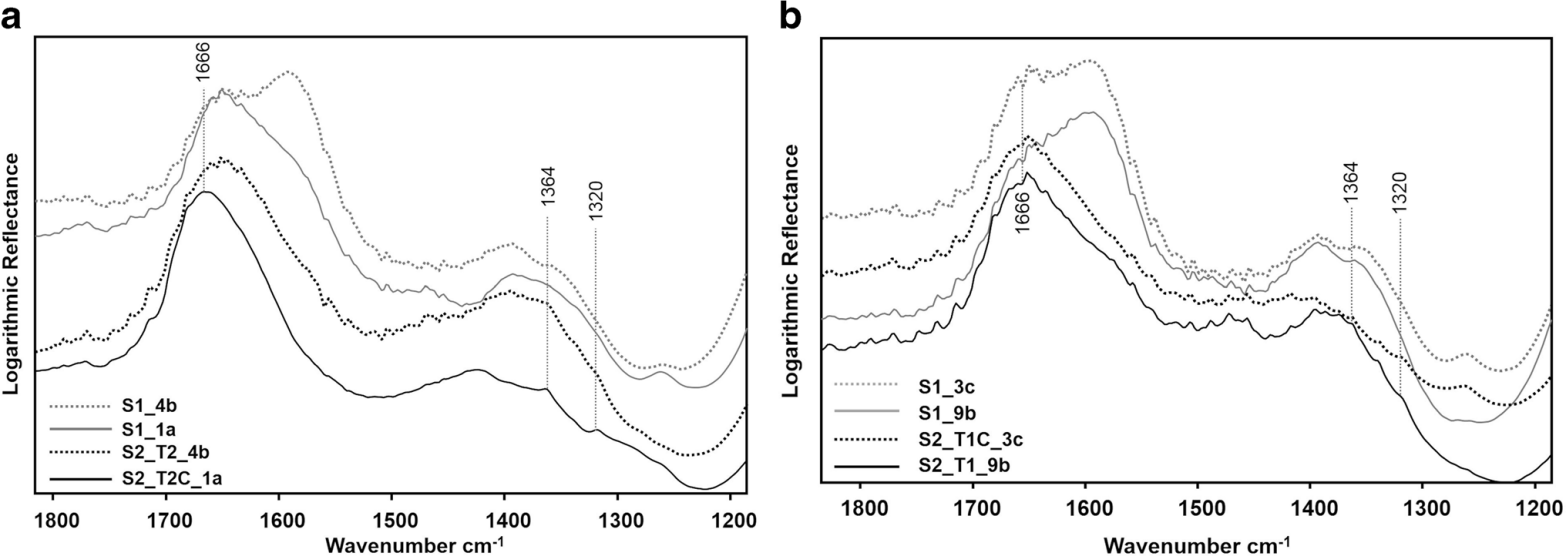
Comparison of TR-FTIR measured on coupons treated with (**a**) T2 and (**b**) T1 methods, before (S1) and after treatment (S2)

The comparison between FTIR spectra acquired on the coupons before and after the treatments (Fig. 10) showed the appearance of weak bands at 1364 and 1320 cm^−1^, as well as the shift of the band at 1655 cm^−1^ towards 1666 cm^−1^, which suggested the formation of moolooite.

**Fig. 11:**
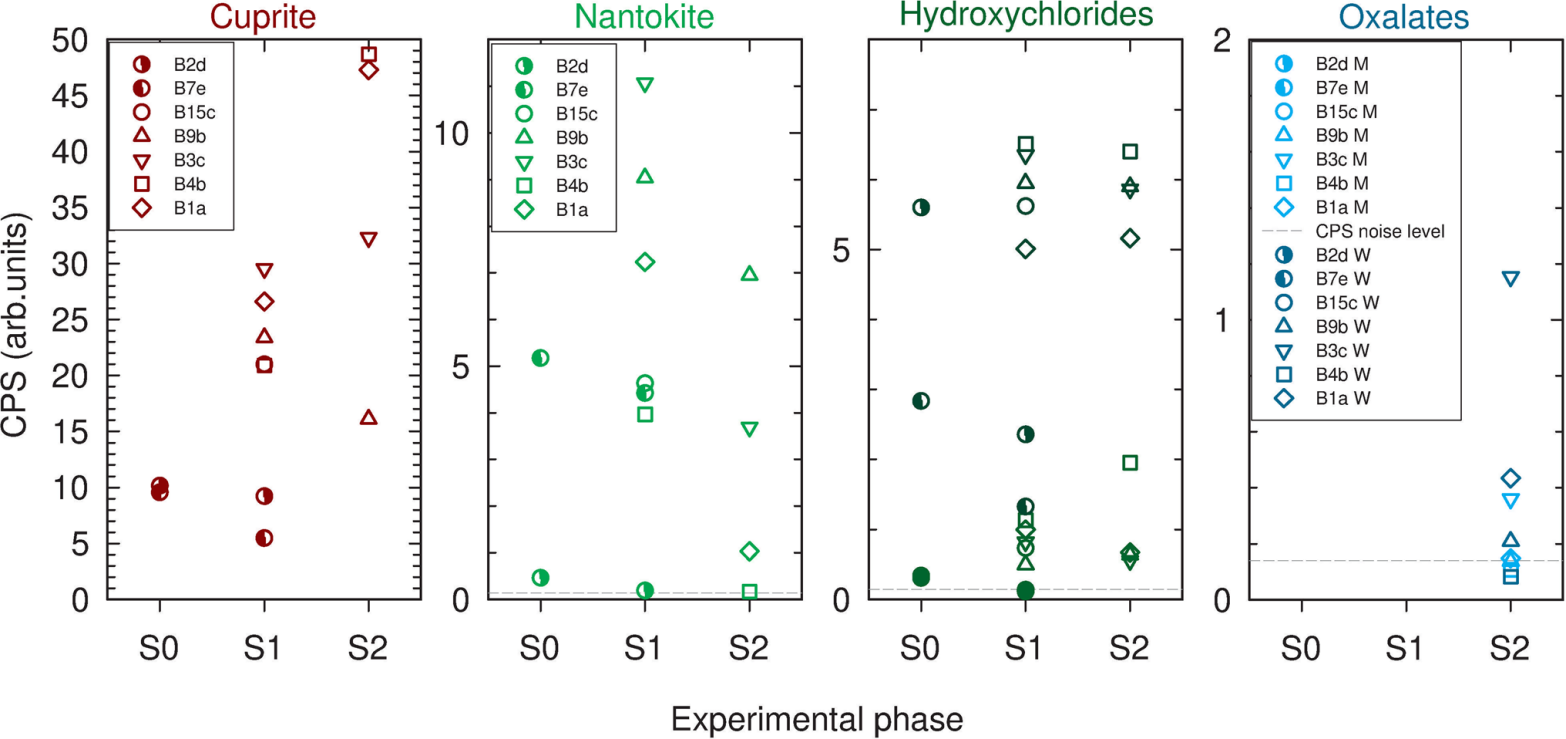
Comparison of XRD monitoring peak counts for the main minerals identified on the selected monitoring areas at the specified experimental phases

The evolution of XRD peak counts on coupons B1, B3, B4, and B9 is shown in Fig. 11. After 8 h of NOX application, a stronger decrease of the nantokite peak intensity on B3 (T1C) than B9 (T1) is measured. The main peaks of copper hydroxychlorides are almost the same and negligible variations of cuprite peak intensity are detected. After a 16-h application of NOX, a very strong decrease on B1 (T2C) and elimination of nantokite main peak on B4 (T2) have been obtained. Significant increase of cuprite is observed, while copper hydroxychlorides are almost the same on both coupons. Wheatleyite is detected on all but B4 (T2), with a much higher intensity on B3 (T1C). On B3 moolooite may also be identified, while on the other measured areas the peak at 22.9° used to monitor moolooite cannot be identified above the noise level. The XRD patterns acquired by means of the diffractometer Bruker New D8 “Da Vinci” are characterised by a much better S/N ratio, and the main peak of moolooite can be distinguished from background on B3c (T1C) and B9b (T1). The overall comparison of XRD results between S1 and S2 phases highlights a general increase of cuprite and a decrease of nantokite as the main result.

The average values of thickness and roughness are reported in Table 4. A thickness around 10 µm and a roughness level around Ra = 2 µm are obtained for all the treatments. The multimodal distributions, detected in phase S1, are no longer identified due to a decrease of values on areas belonging to the S1 clusters with higher values and an increase in the areas belonging to S1 low-value clusters. A slight increase of surface homogeneity can be inferred by the general decrease of standard deviations.

**Table 4:** Average values of thickness (*s*) and roughness (Ra) measured on the selected groups of coupons before (S1) and after (S2) treatments

Group	*s*_S1_	*s*_S2_	Δ*s*_S2-S1_	Ra_S1_	Ra_S2_	ΔRa _S2-S1_
T0	10 ± 4	9 ± 4		2 ± 1	2.0 ± 0.4	
T1	10 ± 4	10 ± 3	0 ± 3	2.0 ± 0.4	1.9 ± 0.4	0.0 ± 0.2
T1C	11 ± 4	10 ± 3	− 1 ± 3	2.0 ± 0.5	2.0 ± 0.4	0.1 ± 0.3
T2	11 ± 3	9 ± 2	− 1 ± 3	2 ± 1	1.9 ± 0.4	− 0.1 ± 0.3
T2C	11 ± 5	11 ± 4	0 ± 2	2 ± 1	2 ± 1	0 ± 1

## Discussion

The preliminary test (see Figs. 1, SI-2, and SI-3) pointed out the easy transformation of nantokite in the tested conditions. Despite the lack of a detailed study of the different reaction paths possibly involved, the tests carried out allowed to put in evidence that superficial nantokite can be easily transformed. Consequently, the effectiveness of treatments to deal with bronze disease should be tested against CuCl trapped in pits inside the patina. This led us to test the coupon weathering procedure described in the “Weathered coupons” section, with the aim of promoting the formation of nantokite trapped inside the patina.

The preliminary test also enlightened the formation of the sodium-copper oxalate wheatleyite (Na_2_Cu(C_2_O_4_)_2_∙2H_2_O) (Rouse et al. 1986) in addition to moolooite. The sodium copper (+ II) double salt wheatleyite is a rare mineral and a water-soluble compound: its formation can be deemed to be out of the objective of forming only insoluble cupric products with the NOX treatment. Wheatleyite is nevertheless a Cu (+ II) product coming from the oxidation of a Cu (+ I) product (presumably nantokite) whose presence, consequently, is reduced. Initially, we thought of rinsing it away at the end of treatment. Moreover, a 3% w/v (COONa)_2_ was adopted in this research instead of a 5% one (Matteini 2015), as the tests suggest a predominance of wheatleyite over moolooite for higher sodium oxalate concentration.

**Fig. 12:**
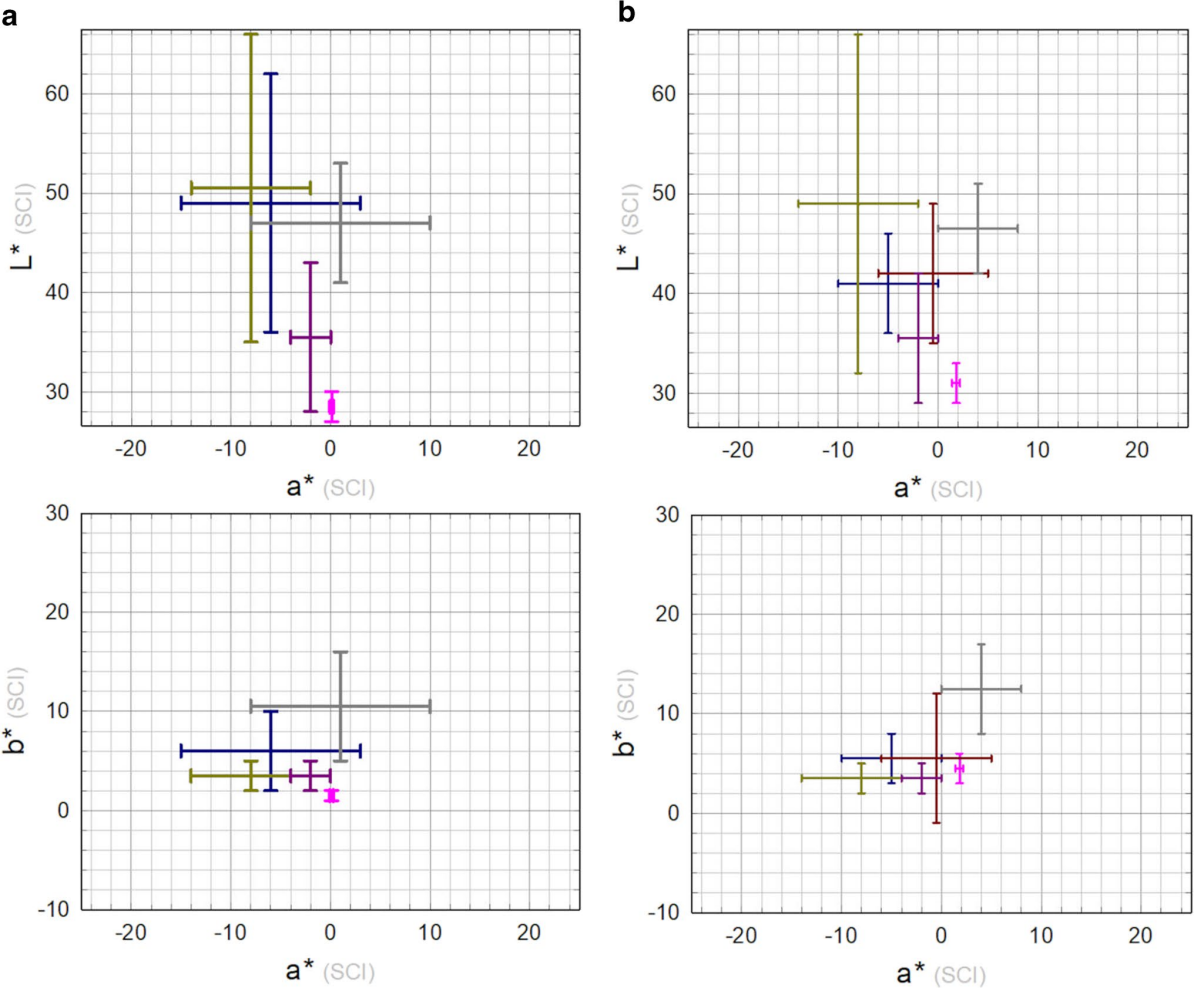
Range of colour values before (**a**) and after (**b**) cleaning measured on coupons in this study (grey) and some monuments (blue, Monumento ai Mille (Franceschi et al. 2006); dark yellow, Brera’s Napoleon (Letardi et al. 2016); pink, Florence Baptistery’s North Door (Letardi et al. 2016); dark red, Neptune Fountain [Salvadori and Letardi, unpublished]; dark pink, Gnecco Tomb’s door (Monari et al. 2021))

The surface properties of outdoor bronze monuments are characterised by a fairly wide variability. This turns out to be a big challenge when more effective protective treatments are to be tested and validated. Weathered coupons along with the set of NDT measurements used for this study allow some comparison with the range of values measured on monuments (Franceschi et al. 2006; Letardi et al. 2016; Monari et al. 2021). The colour range measured on the coupons can be considered similar enough to the ones obtained on a set of different bronze monuments (Fig. 12), with a slight shift towards less green and more yellow hue.

Patina thickness values measured on monuments range from a few tens of micrometres to more than 100 (Letardi et al. 2016; Monari et al. 2021). The patina thickness measured on the coupons is lower, as expected for a corrosion layer developed in less than 2 years; nonetheless, the order of magnitude corresponds to the lower limit measured on monuments. The main chemical composition corresponds to the one that characterises monuments in outdoor marine exposure (Di Carlo et al. 2017). TR-FTIR allowed diagnosing and monitoring of cuprite and copper hydroxychlorides. Nantokite, instead, cannot be identified with this technique due to characteristic wavenumbers outside of the IR spectral range. On the other hand, XRD turns out to be extremely effective at identifying and mapping nantokite both on the coupons’ surface and at the alloy’s interface where the copper salt is mainly present, because of the penetration depth of this technique. Thanks to the grid adopted (Fig. 2), XRD also allowed to show clearly the more localised distribution of nantokite on the coupons. Moreover, a careful repositioning on the same spot before and after treatment allowed a detailed monitoring of the conversion of this compound within patinas.

Due to the large range of variability of the different surface properties on monuments and on the coupons made for this study, an issue to be carefully considered is the proper choice of sampling points and the number of measurements with the different techniques. In fact, the full characterisation of patina thickness on the same 5 points for all the 14 samples allowed to point out a more homogeneous distribution after the selected treatments. A good knowledge of the variability range for the different surface properties also allows to assess how significant changes and differences can be considered among different treatments.

The comparison of measurements before and after cleaning (S1 vs S0) shows a decrease of surface heterogeneities, a clear reduction of the green hue (greater a*), with nantokite spots still present in the patina.

**Fig. 13:**
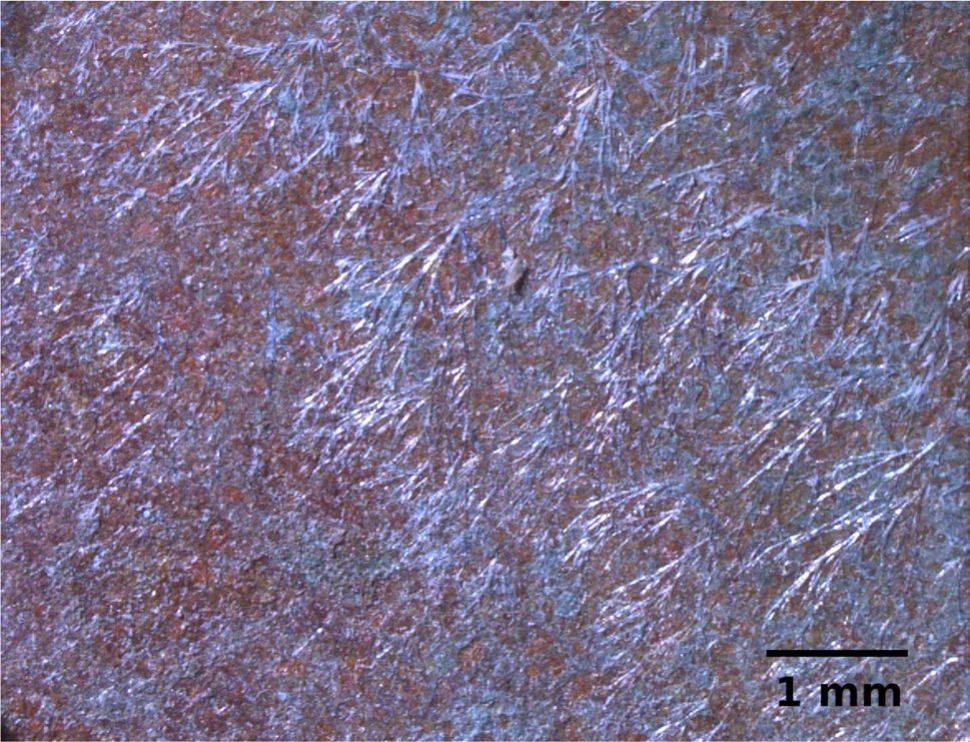
Dino-Lite microscope image on coupon B11 after the treatment T1: typical light bluish needle-shaped microcrystals of Na–Cu oxalate are visible

The number of areas characterised by XRD turned out to be too small to allow statistical analysis of results for the different treatments considered (S2 vs S1), and a larger number of XRD analyses would be advisable in future studies. Nonetheless, a general reduction of nantokite upon treatments can be clearly identified. Moolooite is confirmed as one of the possible products of the sodium oxalate treatment, even though wheatleyite seems more easily formed in our experimental conditions. This sodium-copper oxalate appears as an unaesthetic thin vitreous shiny patina on the surfaces at the end of the treatments, which is formed by light bluish needle-shaped microcrystals when observed under microscope (Fig. 13).

To obtain a more effective removal of this compound from the surface, a second rinsing with deionized spray water was applied on T1 and T2 groups and monitored by stereomicroscope. Several rinsing cycles (average time around 10 s) were alternated to drying. However, only the vitreous shiny patina could be removed, while the undesired whitish stains persisted on the treated surfaces. Indeed, wheatleyite was still identified by XRD on T1- and T2-treated coupons after this additional rinsing, even though with an intensity less than half than on T1C and T2C, respectively, which did not undergo a repeated rinsing. It is worth to underline that the NOX/LW treatment would be followed by the application of a protective coating such as the widely used incralac/waxes that are known to darken the surface.

Before and after the second washing, the thickness and roughness measurements of patinas did not reveal any significant variation.

Both TR-FTIR and XRD confirmed the formation of moolooite on the surface. XRD also detected typical peaks of wheatleyite. Such a different result is maybe due to the greater penetration depth of XRD technique with respect to TR-FTIR. Indeed, it is possible that rinsing with water has solubilised only the most superficial wheatleyite, leaving it in the deepest parts of the patina that are probed by XRD, but not by TR-FTIR.

**Fig. 14:**
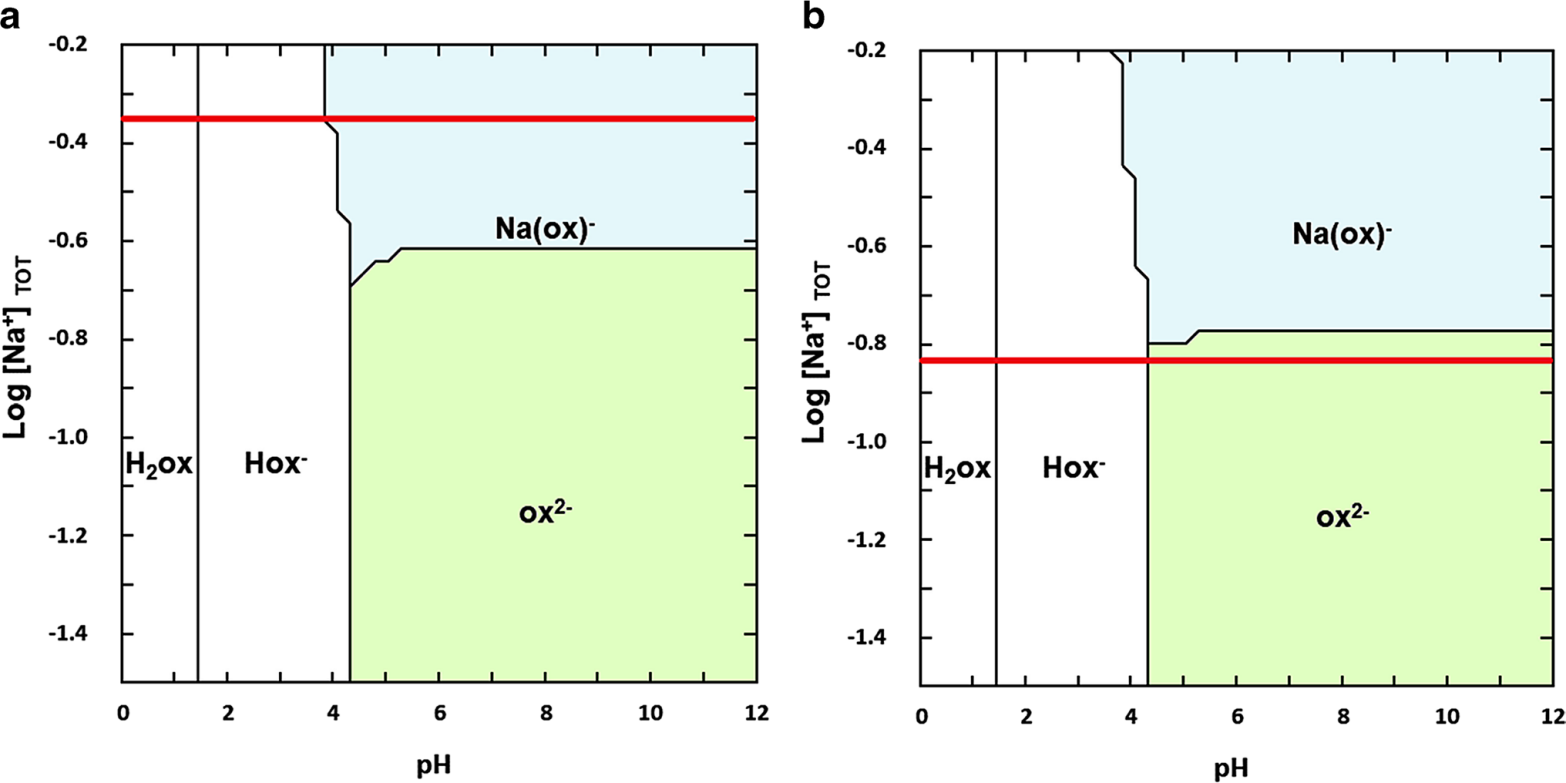
Chemical equilibrium diagrams of Na_2_Ox–H_2_O for 3% w/v Na_2_Ox (**a**) and 1% w/v Na_2_Ox (**b**). The red line marks the log[Na^+^] value corresponding to the total amount of Na_2_Ox in solution. Diagrams were obtained by the Hydra and Medusa software (Puigdomenech 2006)

For a better understanding of wheatleyite formation conditions, the behaviour of the (COONa)_2_ in aqueous solution was examined with the free software Hydra and Medusa developed by KTH (Royal Institute of Technology, Stockholm) (Puigdomenech 2006). The predominance area diagrams are computed at 3% w/v (0.22 M) and 1% w/v (0.07 M) of (COONa)_2_ (Fig. 14). The comparison of the two chemical equilibrium diagrams indicates how the two anions C_2_O_4_^2−^ (Ox^2−^) and NaC_2_O_4_^−^ (Na(Ox)^−^) tend to be produced in aqueous solution according to the sodium oxalate molarity and pH. At neutral pH, with Log [Na^+^] =  − 0.35 (3% w/v Na_2_Ox) indicated by the red line, the formation of the Na(Ox)^−^ species is preferential, favouring the reaction with Cu(II) to produce wheatleyite. At the same pH value, with Log [Na^+^] =  − 0.83 (corresponding to 1% w/v (COONa)_2_), the formation of Ox^2−^ is predominant, favouring the formation of moolooite. In conclusion, the results seem to suggest using a less-concentrated solution for a better inhibitor effectiveness.

## Conclusions

We focused our attention on some methodological issues in order to better characterise the effectiveness of conservation treatments against bronze disease.

The surface properties of foundry (cast) coupons weathered at GEMS about 18 months showed a good degree of similarity with the range of values measured on several outdoor bronze monuments. Moreover, they are characterised by a hidden and uneven distribution of nantokite that can provide a good mock-up to test treatments to inhibit bronze disease on the complex and uneven surfaces of outdoor artworks.

A well-defined measurement grid to compare results before and after treatments with a set of non-destructive techniques on the same area turns out to be an effective tool to deal with the large variability of surface properties that characterise outdoor bronze patinas. Moreover, a statistical approach is suggested as the more effective way to characterise and compare the overall behaviour of treatments to be adopted in the conservation of outdoor bronze artworks.

XRD measurement directly on the corroded bronze surface is the most useful technique to monitor in a non-destructive way the local presence and conversion of nantokite in the bronze patina. A more refined analysis of XRD patterns on a well-defined measurement grid would allow deepening the knowledge on the bronze disease treatment effectiveness and optimisation.

The tests performed suggest the reduction of nantokite with the sodium oxalate treatment. The even small emergence of moolooite in the patina is an encouraging sign of the effectiveness of the NOX-LW treatments against bronze disease. Nonetheless, a more detailed study to optimise parameters such as (COONa)_2_ concentration would be advisable. A more detailed study on treatment penetration depth and its ability to seal the surface against possible re-activation of bronze disease would be of utmost interest for practical applications. Moreover, the performance of the treatment upon weathering should be investigated and compared with inhibitors currently in use.

## Supplementary Information

Below is the link to the electronic supplementary material.

Supplementary file1 (PDF 1261 KB)

## Data Availability

The datasets generated and analysed during the current study are available from the corresponding author on reasonable request.
