# Supplementary material for: Protective treatments for copper alloy artworks: preliminary studies of sodium oxalate and limewater effectiveness against bronze disease
Source: Environ Sci Pollut Res Int. 2022 Nov 16;30(10):27441–57. doi: 10.1007/s11356-022-24107-0 (PMC9995518; doi:10.1007/s11356-022-24107-0)
Supplement: Supplementary file 1 — Supplementary file1 (PDF 1261 KB) [file 11356_2022_24107_MOESM1_ESM.pdf]

Supplementary Information

Protective treatments for copper alloys artworks: preliminary studies of sodium oxalate and lime water effectiveness against bronze disease

Giulia Monari[1], Monica Galeotti[3], Mauro Matteini[4], Barbara Salvadori[2], Roberto Stifanese[1], Pierluigi Traverso[1], Silvia Vettori[2], Paola Letardi[1*],

1 CNR-IAS via De Marini 16, Genova, Italy

2 CNR-ISPC via Madonna del Piano 10, Sesto Fiorentino, Italy

3 OPD Viale F. Strozzi 1, Firenze Italy

4 Conservation Scientist, Florence, Italy

* Corresponding author

paola.letardi@cnr.it, +39 347 2765957

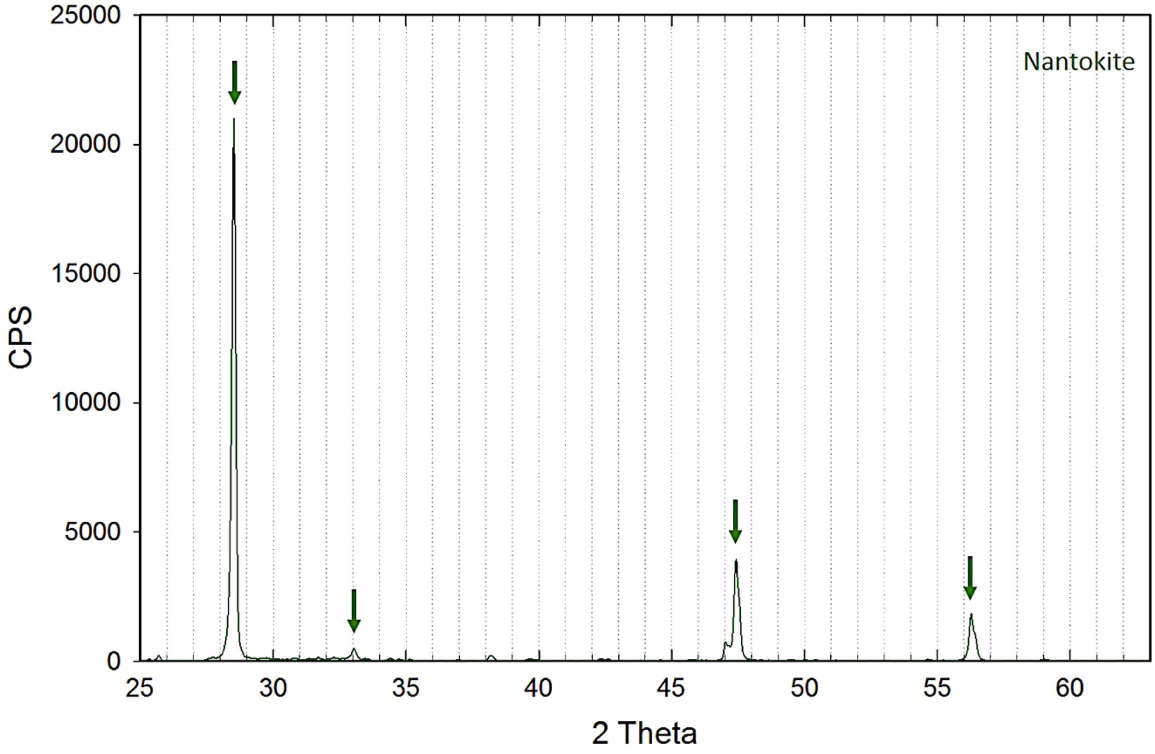

Fig SI-1. Powder XRD diffractogram obtained on syntetised nantokite

a

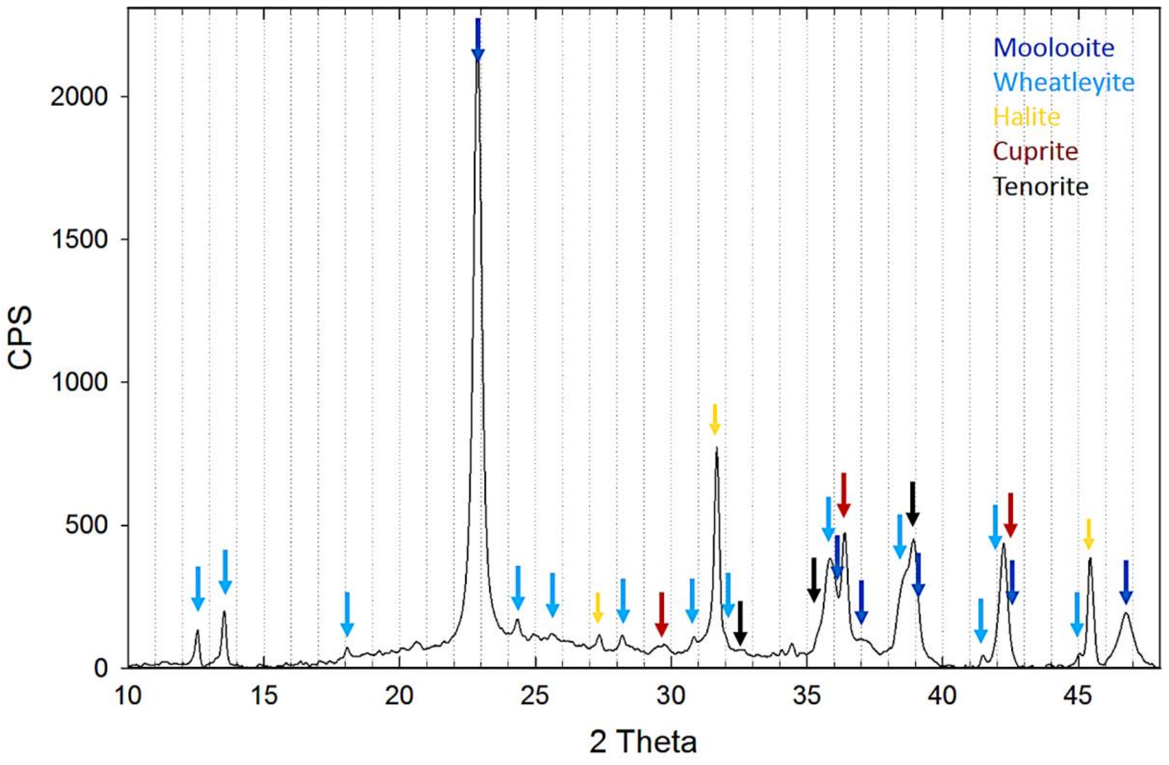

b

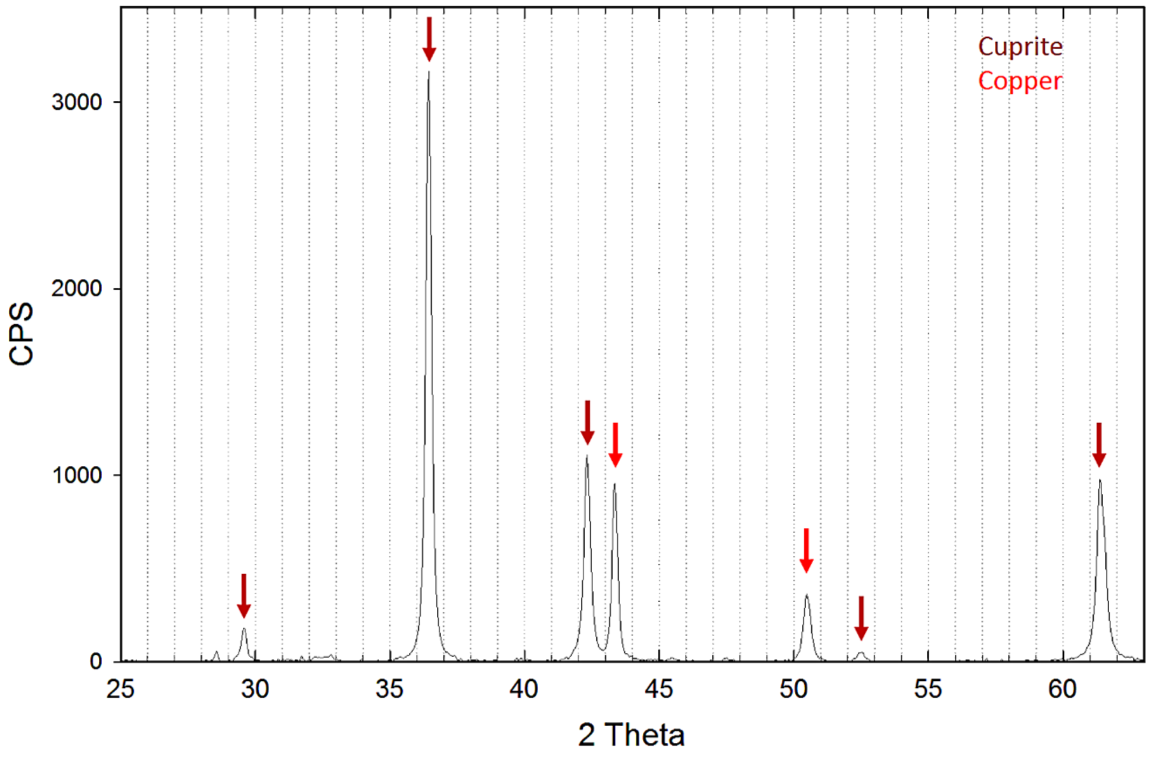

Fig SI-2. Powder XRD diffractograms on precipitated products obtained in aqueous solution: a) addition of sodium oxalate to a nantokite saturated solution; b) addition of nantokite to a sodium oxalate saturated solution;

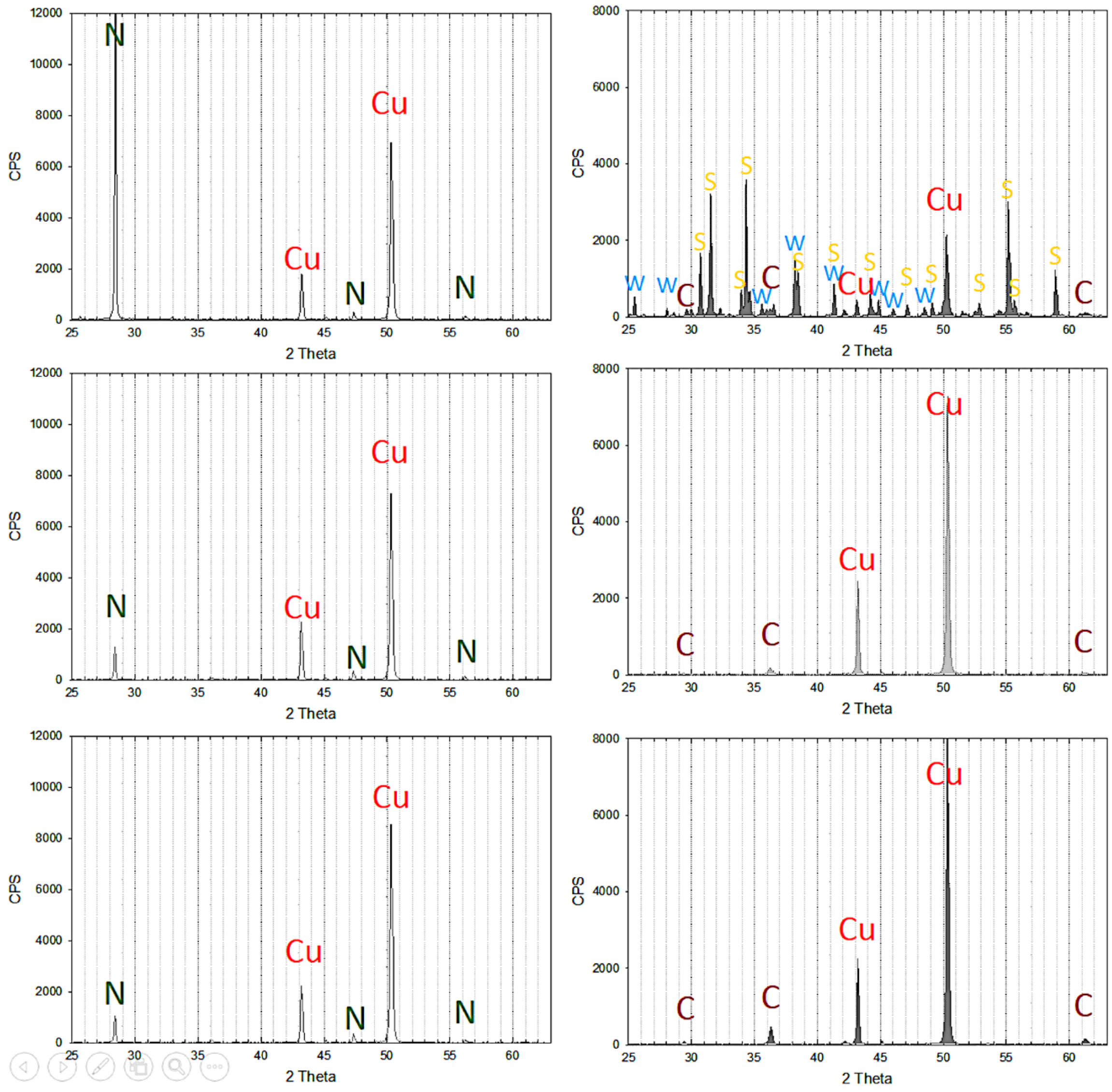

Fig SI-3. XRD diffractogram obtained on copper coupons with syntetic nantokite before (left) and after (right) treatment. First row: sodium oxalate poultice (a); second row: immersion in a 5% w/v sodium oxalate solution (b); third row: immersion in deionised water. Peaks label are as follows: N-nantokite, Cu-copper, C-cuprite, S-sodium oxalate, W- wheatleyite.
